# *Theobroma* spp. Mucilage as a Valuable Natural Ingredient: Composition, Potential for Food Innovation, and Future Perspectives

**DOI:** 10.3390/foods15020185

**Published:** 2026-01-06

**Authors:** Frankdux Reynaldo Huanca-Ccompe, Hilka Mariela Carrión-Sánchez, Lucero Quispe Chambilla, Sylvia Carolina Alcázar-Alay, Augusto Pumacahua-Ramos

**Affiliations:** Department of Food Engineering, Universidad Nacional Intercultural de Quillabamba, Cusco 08741, Peru; hilka.carrion@uniq.edu.pe (H.M.C.-S.); lucero.quispe@uniq.edu.pe (L.Q.C.); augusto.pumacahua@uniq.edu.pe (A.P.-R.)

**Keywords:** *T. cacao*, *T. grandiflorum*, *T. bicolor*, exudate, cocoa honey, by-product, functional ingredient, theobromine, catechins, reducing sugar

## Abstract

Peru is currently distinguished by its remarkable biodiversity, which is characterized by a high level of endemism and a wide array of ecological niches. In the context of biodiversity, the genus *Theobroma* spp. is particularly noteworthy, encompassing the species *Theobroma cacao*, *Theobroma grandiflorum* and *Theobroma bicolor,* which are collectively referred to as cacao, cupuaçu, and macambo, respectively. The primary economic value of these species is derived from their mucilage-rich pulp and beans. In recent years, the mucilage of the genus *Theobroma* has gained economic relevance due to its flavor, floral and fruity aroma. The present review article aims to provide a comprehensive exploration of *Theobroma* spp. mucilage, addressing its characterization and potential applications. The present study investigates aspects related to its origin, cob morphology, proximal composition, bioactive compounds, volatile profile and its application in the food industry. The study highlights a high content of polysaccharides such as reducing sugars, organic acids, pectin, cellulose, hemicellulose, antioxidant capacity, presence of polyphenols and methylxanthines. Through this comprehensive review, a prospective vision is proposed on the opportunities for innovation and sustainable development around the *Theobroma* mucilage industry, highlighting its relevance not only as a agri-food byproduct, but also as a valuable resource in the productive circular economy and the sustainability of biodiversity.

## 1. Introduction

The Peruvian Amazon region has a lot of different plants and animals. One important group is the Malvaceae family, which includes 243 types and 4300 species [1]. This endemic family contains the genus *Theobroma* spp., which is characterized by its multiple applicability of grains in the agro-industrial and pharmaceutical sectors [2]. The genus Theobroma comprises 20 species, nine of which are found in the Amazonian region. The most notable species are *T. cacao*, *T. grandiflorum* and *T. bicolor* [3]. Although the trees have similar morphologies; their flowers and fruits differ in shape and colour [4]. The pods of the *T. cacao* tree can vary from having prominent grooves to being almost smooth, and display colors such as bright green, dark green, yellow or dark red, or a combination of these colors [5]. Conversely, the fruits of *T. grandiflorum* range in colour from light brown to dark brown, and can be oblong or ellipsoidal, and its seeds are covered by a semi-liquid mucilage that is ivory-colored [6,7]. The fruit of *T. bicolor* is ribbed, grooved and veined; its seeds are covered with creamy, yellowish mucilage [8].

*Theobroma* seeds are a significant source of polyphenolic compounds and methylxanthines [9]. Despite their phylogenetic proximity, flavonoid compounds unique to *T. cacao*, such as theograndins, are detected in *T. grandiflorum* [10,11]. Several studies have demonstrated the high antioxidant capacity of the exuded fermentation residues and the presence of compounds such as catechin, epicatechin and procyanidin [8,12,13]. The mucilages of the genus *Theobroma* are a source of nutrients and photochemical bioactives, including phenolic compounds. They are a promising source of alternative food ingredients for use in various sectors of the food, pharmaceutical and other industries.

The present review article aims to demonstrate the composition, functional properties and potential applications of mucilage from three species of *Theobroma* in order to stimulate interest among researchers in their reintroduction to the food industry using a circular economy approach.

## 2. Methodology

The present review has been proposed from an overview of *Theobroma* spp. Specifically, this review presents information on *T. cacao*, *T. grandiflorum* and *T. bicolor* mucilage, collected from scientific literature in the Scopus, ScienceDirect, SpringerLink and Web of Science databases. A comprehensive search was conducted using a range of keywords, including ‘mucilage’, ‘pulp’, ‘exudate’, ‘cocoa honey’, ‘bioactive compounds’, ‘volatile compounds ‘and ‘industrial applications’, all including ‘*Theobroma* spp.’ term.

Using the keyword ‘*Theobroma* spp.’, the search range was set to between 2000 and 2025, showing approximately 1150 results. The search was then refined using specific keywords, reducing the number of results to around 130 works. Around 60% of the articles reviewed were included in this review.

The data were systematically organized for the purpose of writing the present article, with a particular emphasis on current evidence and studies that were supported by scientific methodology.

### 2.1. Geographical Distribution of Theobroma spp.

**Figure 1:**
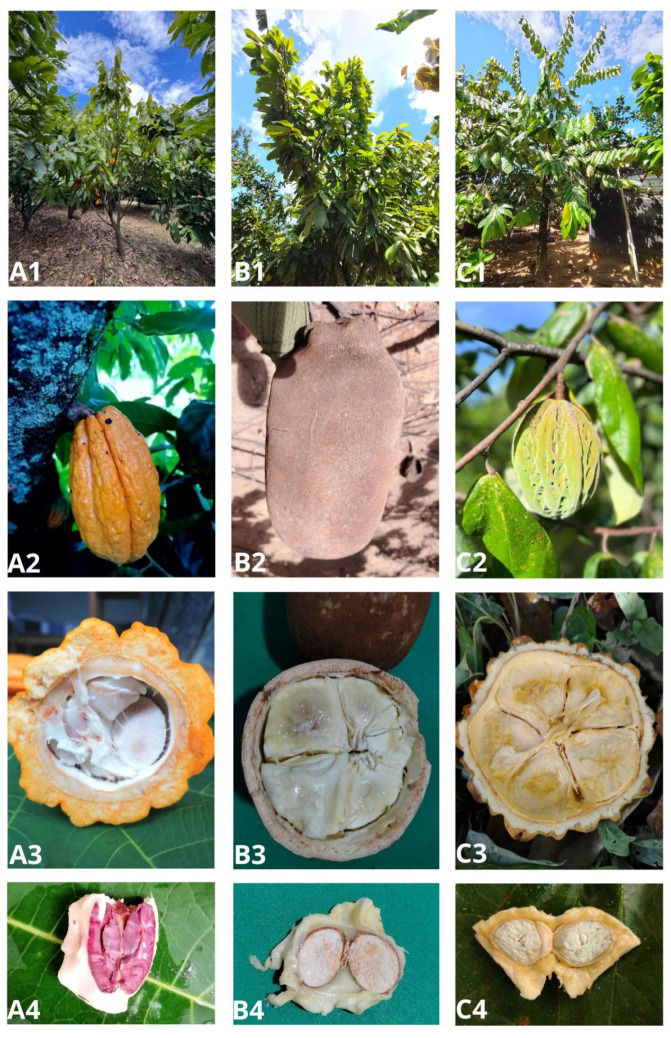
Main species of the genus *Theobroma spp*. (**A**) *T. cacao*, (**B**) *T. grandiflorum* and (**C**) *T. bicolor.* Photographic views from the tree (**A1**,**B1**,**C1**), cob (**A2**,**B2**,**C2**), cross-section of cob (**A3**,**B3**,**C3**), grains and mucilage (**A4**,**B4**,**C4**).

*Theobroma* spp. species are geographically distributed across various tropical regions of the world. They are a vital agricultural resource for communities in Latin America and other tropical regions of Asia and Africa. Figure 1 presents the main species of the genus *Theobroma*, specifically *T. cacao*, *T. grandiflorum*, and *T. bicolor*, and includes representative views of the tree, the cob, internal components of the cob, as well as the mucilage covered grains.

In *T. cacao*, studies indicate that the greatest genetic diversity is found in the humid forests of the Amazon region [14,15]. Within cacao species, two important groups have been identified: the Criollo and Forastero. The classification of these groups is based on distinct morphological characteristics, including the size and thickness of the cob, the mucilage/grain ratio, and other characteristics. However, the process followed by the chocolate industry is analogous, start with the harvesting of the pods, followed by partial separation of the mucilage from grains and, consequently fermentation. The process ends with the drying and roasting of the grains [16].

*T. grandiflorum* has also been documented as originating in the Amazon region [17], with a predominance of occurrence in the northern part of Brazil. This species has contributed to agriculture in Central America, South America and some Caribbean islands, especially in countries such as Peru and Brazil [18]. This Amazonian fruit has been classified as highly commercially viable due to its intense and pleasant aroma, exotic flavour and creamy texture. These sensory characteristics make it ideal for consumption fresh and for use as an ingredient in the preparation of different food products, such as ice cream, natural juices, jams, yoghurts and compotes [19,20].

*T. bicolor*, more commonly referred to as “macambo” or “white cocoa”, is a species that demonstrates a propensity for growth in warm, humid tropical environments. The geographical origins of this practice remain a subject of debate, with some attributing its origins to Central America, while others suggest South America as the more probable origin [1]. The production is geographically diverse, extending from Mexico to northeastern Brazil, including countries such as Colombia, Venezuela, Ecuador and Peru. Clearly, *T. bicolor* plant has been domesticated and refined technologically for the purpose of producing seeds and mucilage. Its products are destinated for use in the food and cosmetic industries [1]. Table 1 presents general information on the production and morphology of the plants and characteristics of the mucilage.

### 2.2. Agro-Industrial Byproducts from the Processing of Theobroma spp.

The cob, which is the fruit of *Theobroma* spp. tree, is composed of an outer shell, the inside of which contains beans surrounded by a pulp known as mucilage [27], which is released mainly during the opening of the ear and fermentation. Table 2 shows the percentages of agro-industrial byproducts generated from the processing of *T. cacao*, *T. bicolor* and *T. grandiflorum.*

**Figure 2:**
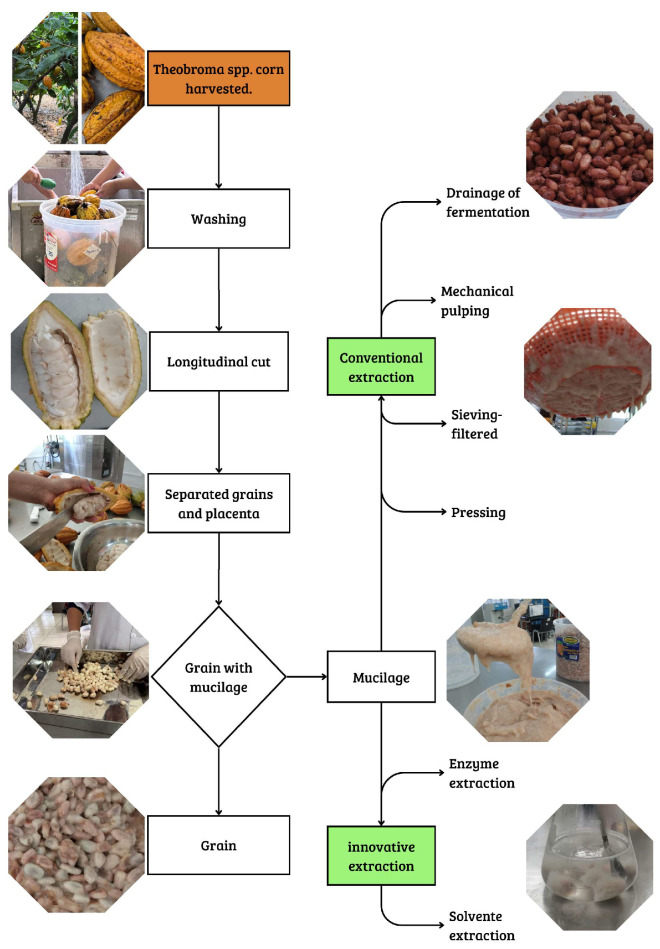
Conventional methods and innovative strategies for the extraction of *Theobroma spp.* mucilage.

The extraction of *Theobroma* spp. mucilage can be carried out using traditional methods, based on physical separation as natural drainage during fermentation [12], mechanical pulping [30,31], sieving–filtering [32,33,34] and pressing [35]. However, innovative methods have been proposed, including extraction using organic solvents such as water, ethanol, and methanol, as well as pectolytic enzyme-assisted extraction through liquefaction processes, applied to the mucilage of *Theobroma* spp. after the pulping stage, with the aim of facilitating the breakdown of the polysaccharide matrix and improving the efficiency of exudate recovery [30,36]. Figure 2 illustrates the conditioning process of *Theobroma* spp. mucilage-coated grains, as well as the separation methods employed.

In general, byproducts generated during the processing of cobs are not adequately utilized, which represents a significant loss of biomass. In this context, it is essential to highlight the chemical composition, phytochemistry and volatile profile, with the aim of reintroducing it into the food industry and the sustainability of the supply chain. Despite its great potential for human consumption, *Theobroma* spp. mucilage is still scarcely exploited or studied.

## 3. Mucilage

Mucilage is a semi-liquid substance found in the family Plantaginaceae, Malvaceae Liliaceae, Linaceae, Plantaginaceae and Cactaceae, specifically in roots, bulbs, tubers, flowers and leaves [37,38]. *Theobroma* spp. mucilage is a complex, water-soluble polysaccharide, consisting mainly of monosaccharides and organic acids linked by glycosidic bonds, as well as glycoproteins and other compounds [39,40,41].

Recent research considers mucilage, in generic families, as a hydrocolloid containing arabinogalactan-proteins (93.2–98.2%), in which the protein fraction is linked to the carbohydrate chain [42,43].

*Theobroma* spp. mucilage plays a crucial role in the grain fermentation process, acting as a substrate for yeasts and bacteria (lactic and acetic). These microorganisms convert mucilage into leachates [44,45]. However, previous studies suggest partial pulping of the grains without negatively affecting the natural fermentation of the grains and their final sensory quality [46,47,48,49].

### 3.1. Chemical Constituents of Mucilage Theobroma spp.

Given its nature, *Theobroma* spp. mucilage is rich in simple sugars, polysaccharides, organic acids, phenolic and volatile compounds, making it a byproduct of great interest for the development of functional foods [12,50,51,52,53,54]. The chemical composition of the mucilage of *T. cacao*, *T. grandiflorum* and *T. bicolor* has been the subject of many studies. Table 3 shows the proximal macromolecular composition of the mucilage, as well as the physicochemical properties, reducing sugars and bio-compounds of the three species studied.

These compositional differences have direct implications for the potential applications of *Theobroma* spp. mucilage. The high water content (82–93%) and soluble solids values (12–16 °Bx) are related to the carbohydrate content. In particular, *T. cacao* exhibits the highest °Bx content compared to the other two species [8]. Furthermore, an evaluation of the physical and chemical properties of the three species revealed that *T. cacao* and *T. grandiflorum* exhibited relatively acidic pH values (3–4). In contrast, *T. bicolor* exhibited a pH slightly acidic to near neutral (5–7) [8,54]. These values correlate with titratable acidity-TEA, expressed as citric acid, with contents of 60, 18, and 8 mg/g dry weight (DW), respectively, which manifests as a sweet-sour taste [8].

With regard to reducing sugar content, *T cacao* has the highest levels, followed by *T. bicolor* and *T. grandiflorum*. The sugar profile indicates a high concentration of sucrose in *T. grandiflorum* and *T. bicolor*. *T. cacao* has higher concentrations of glucose (69 mg/g) and fructose (72 mg/g), but these fermentable sugars contribute to the fermentation process carried out by *S. cerevisiae*, which transports and metabolizes them directly through its glycolytic pathways [58].

It has been demonstrated that *T. cacao* mucilage is characterized by the present of pectin as a structural polysaccharide, ranging from 0.5 to 1.2%. Research conducted by [64] involved the extraction, purification, and quantification of pectin from the mucilage of *T. grandiflorum*, revealing a high content of galacturonic acid. The pectin was mainly composed of highly esterified homogalacturonan, with a low degree of acetylation, as well as small regions of rhamnogalacturonan containing side chains rich in galactose and arabinose. However, increases in temperature and the activity of pectolytic enzymes, such as endopolygalacturonase, contribute to the degradation and liquefaction of these pectins during processing and fermentation [56].

Regarding the fatty acid profile of mucilage from *Theobroma* species, *T. cacao* has been reported to contain a low total lipid content (0.8–1.5%), although a detailed fatty acid profile for its mucilage has not yet been described [59]. In contrast, *T. grandiflorum* mucilage has been reported to be dominated by palmitic acid (55.22%), oleic acid (18.8%), and α-linolenic acid (17.9%) [60]. *T. bicolor* exhibits a higher proportion of saturated fatty acids, mainly stearic acid (49.6%) and palmitic acid (6.1%), whereas unsaturated fatty acids are primarily represented by oleic acid (39.9%) and linoleic acid (2.1%); the latter have been associated with protective mechanisms against fruit oxidation [65].

In this context, based on proximate quantification, physicochemical and structural characteristics, and micronutrients, the mucilage of *Theobroma* spp. presents a compositional profile that supports its potential incorporation into various lines of the food industry. However, the differences observed in its composition can be attributed to geographical variability, fruit maturity stage, harvest season, and intraspecific genetic variations [10].

From a food safety perspective, it is relevant to consider the potential presence of allergenic compounds in *Theobroma* spp. mucilage. Although this byproduct has traditionally been regarded as a safe food ingredient [66], the presence of pathogenesis-related (PR) proteins, including chitinases, osmotins, and β-1,3-glucanases, has been reported in both the mucilage and the testa tissues of cacao beans [8,67]. These enzymes play a key role in plant defense mechanisms by acting as antifungal regulators through the inhibition of spore germination and the degradation of fungal cell wall components. In particular, chitinases have been associated with cross-reactivity in latex-sensitized individuals, a phenomenon known as latex–fruit syndrome [68]. Meanwhile, osmotins and β-1,3-glucanases have demonstrated the ability to induce immunoglobulin E (IgE) synthesis, thereby triggering allergic responses in susceptible individuals [69].

### 3.2. Bioactive Compounds in Theobroma spp.

Bioactive compounds are phytochemical molecules naturally present in food or agro-industrial byproducts. Although they are not considered essential nutrients, they have the ability to interact with one or more constituents of living tissues, thus exerting relevant physiological effects [70]. These compounds exhibit diverse biological activities, including antioxidant, anti-inflammatory, antimicrobial and immunomodulatory properties, which explains the growing interest in the field of health and disease [71,72].

Species of the genus *Theobroma* spp. contain a wide variety of functional bioactive compounds with antioxidant activity, present in both the grain and mucilage, particularly in the form of flavonoids and methylxanthines and organic acids [10,60,73]. *Theobroma* grain is one of the main sources of these compounds; however, its use is limited by its low stability against oxidation and thermal degradation processes [9]. These compounds are primarily located in the cotyledons of the grains and can be lost during processing due to diffusion into the surrounding environment. Consequently, a significant fraction of the compound of interest can be transferred to the husk ant exudate, leading to the generation of byproducts with greater functional value rich in flavonoids and methylxanthines [11], procyanidins and proanthocyanidins [13], catechin and epicatechin extracts [74]. Table 4 provides a comprehensive overview of the compounds of interest present in mucilage, the processing parameters and the analysis method.

#### 3.2.1. Bioactive Compounds

The main bioactive compounds reported in the mucilage of *Theobroma spp*. have been quantified primarily using UV-Vis spectrophotometry techniques, with the Folin–Ciocalteu method being the most widely used for determining total polyphenols. Available studies show a higher total polyphenol content in *T. bicolor* (40–245 mg GAE/100 g), followed by *T. cacao* (50–105 mg GAE/100 g) and *T. grandiflorum* (40–66 mg GAE/100 g). The quantification of total flavonoids in the mucilage of *T. cacao* and *T. grandiflorum* has also been reported, with values ranging from 5.16 to 36.80 mg/100 mL and from 0.6 to 4 mg QE/100 g, respectively.

Quantification of bioactive compounds by high-performance liquid chromatography (HPLC) confirmed the presence, in the mucilage of *T. cacao*, of flavonoids belonging to the catechin and epicatechin (0.95 mg/100 g) and proanthocyanidins B1, B2 and C1 (2.07 mg/100 mL), as well as xanthines, including theobromine in the range of 0.49 to 2.66 mg/100 mL and caffeine between 0.12 and 0.91 mg/100 mL. Several studies have reported a reduction in these alkaloids during the fermentation of the cocoa bean, which has been associated with their possible diffusion along with the exudated cellular fluids, released as a consequence of the increased permeability of the testa [51,80,81]. Furthermore, this technique has shown the presence of phenolic acids, such as gallic acid, 4-hydroxybenzoic acid and m-coumaric acid, in the mucilage of *T. bicolor* [54].

#### 3.2.2. Antioxidant Capacity

The antioxidant capacity of *Theobroma* spp. mucilage has been evaluated using ABTS, FRAP, ORAC, and DPPH assays across the three species studied. Using the ABTS method, antioxidant activity values ranged from 4.5 to 8.5 µM TE/mL in *T. cacao*, from 90.6 to 96.9 µM QE/g in *T. grandiflorum*, and reached a value of 3.3 in *T. bicolor*. In contrast, FRAP-based antioxidant activity has only been reported for *T. cacao*, with values between 3.35 and 7.89 µM TE/mL. Regarding the ORAC assay, values of 1.28–1.33 µM TE/mL were observed in *T. cacao*, whereas significantly higher values (50.22–67.97) were reported for *T. grandiflorum*. Additionally, DPPH scavenging activity values of 4.09–4.11 µM TE/g were reported for *T. grandiflorum*, while a value of 1.93 mg TE/100 g was reported for *T. bicolor*. Importantly, several studies have demonstrated a positive correlation between total polyphenol and flavonoid contents and the antioxidant activity of cocoa mucilage [12].

In this context, *Theobroma* spp. mucilage, when classified according to the antioxidant effects of its bioactive compounds, exhibits a relatively high value compared to byproducts such as shell and testa, although it shows a lower antioxidant content than the cocoa bean. According to [82], who evaluated the antioxidant capacity of foods using the FRAP method, fruits and fruit juices present an average value of 0.69 mmol/100 g. Based on this criterion, *Theobroma* spp. mucilage can be categorized as a food with medium to high antioxidant capacity.

The compiled literature shows that botanical factors such as genetics, geographic origin, and maturity stage contribute substantially to the bioactive variability of *Theobroma* spp. mucilage [52]. Independently, factors such as the analytical method, processing conditions, and storage (fresh, frozen, and freeze-dried) can promote undesirable chemical and enzymatic reactions that affect the final quantification [75,83]. Furthermore, extraction parameters such as temperature, extraction time [84] and solvent polarity [78] have been shown to significantly influence both the overall extraction yield and the profile of the recovered compounds.

Although significant levels of bioactive compounds have been reported in the mucilage of *Theobroma* spp., there is currently no direct clinical evidence to support their benefits for human health. However, several in vitro studies have demonstrated that the intake of phytochemicals at low concentrations exhibits antioxidant and anti-inflammatory properties, which contribute to the prevention of cell damage, skin aging, and certain types of cancer, thus benefiting health [85]. These findings suggest that the compounds present in mucilage have high functional potential.

#### 3.2.3. Antimicrobial Activity

Specific studies related to the antimicrobial activity of *Theobroma* spp. mucilage demonstrated no inhibition. However, non-traditional mucilages such as *T. subincanum* have shown a positive bacterial inhibitory effect against *Staphylococcus aureus* and *Streptococcus mutans*, with a minimum inhibitory concentration (MIC) of 37.5 mg/mL for both bacteria [54]. On the other hand, components of *Theobroma* spp. showed antimicrobial potential, such as the ethanolic extract of *T. cacao* beans against *S. mutans*, which presented inhibitory zones ranging from 6–15 mm, being less effective than chlorhexidine [86]. *T. bicolor* beans showed inhibition against the fungus *C. albicans*, with an MIC of 75 mg/mL. Similarly, aqueous and methyl extracts of cocoa leaves showed a positive effect against *Staphylococcus aureus* and *Escherichia coli* with inhibition zones of 12.00–26.33 mm and 14.33–26.67 mm, respectively, with a concentration range of 100 to 500 mg/mL [87]

### 3.3. Volatile Compounds in Theobroma spp.

*Theobroma* spp. is characterized by an exotic flavour, which is valued for its organoleptic properties, emphasizing the presence of floral and fruity volatile compounds [88]. As demonstrated in the studies conducted by [89,90], the volatile composition of mucilage is susceptible to influence from various factors, including genotype, cob origin and degree of maturity. Conversely, the extraction of these compounds has been achieved through the utilization of techniques such as simultaneous distillation–extraction, headspace and solid-phase extraction, followed by analysis via gas chromatography/mass spectrometry/olfactometry (GC/MS/O) [38]. As illustrated in Table 5, the results from various studies on the primary volatile components are reported.

It has been observed that *T. cacao* mainly presents aromatic regions dominated by aldehyde compounds, followed by carboxylic acids, lactones, phenols, and ketones, which vary according to geographic origin, genotype, and conditioning. Studies conducted by [31] characterized cocoa from Indonesia, Vietnam, Cameroon, and Nicaragua, highlighting key odorants such as trans-4,5-epoxy-(E)-decenal, 2- and 3-methylbutanoic acid, 3-(methylthio)propanal, 2-isobutyl-3-methoxypyrazine, (E,E)-2,4-nonadienal, (E,E)-2,4-decadienal, 4-vinyl-2-methoxyphenol, δ-decalactone, 3-hydroxy-4,5-dimethylfuran-2(5H)-one, dodecanoic acid, and linalool. Complementarily, Ref. [22] identified 2-pentanal, linalool, 2-pentanone, and 2-methyl-3-buten-2-ol in Chuncho cocoa cultivars, associating these compounds with sensory descriptors such as almond, floral, fruity, and herbal. In agreement, Ref. [95] reported the presence of phenolic odorants, including 4-vinyl-2-methoxyphenol, methylphenol, and coumarin in the exudate, as well as the active contribution of linalool to aromatic complexity during the fermentation process, highlighting the possible diffusion of mucilage into the bean. Studies [31,55,91,96] have identified the presence of 2-phenylethanol in fresh mucilage, a compound widely recognized as “the flavor molecule” due to its commercial value and extensive use in the food industry. However, the presence of undesirable compounds such as indole characterized by intense floral notes accompanied by fecal nuances has also been reported. Indole can be reduced through the application of thermal treatments, thereby contributing to an improvement in the final sensory profile of the product, as well as to the formation of compounds belonging exclusively to the phenol and methylphenol groups (fruity and floral aromas) resulting from the degradation of polyphenols, lignin, enzymes, and microorganisms [55].

With regard to *T. grandiflorum* mucilage, the predominant major volatile components are found to be from the ester group, followed by terpenes and alcohols, such as ethyl butanoate, ethyl hexanoate, and linalool [19]. These esters have also been reported at predominant concentrations by [92,97], whereas Ref. [98] reported linalool and its oxides as an important fraction of compounds released through enzymatic hydrolysis. On the other hand, Ref. [19] compared acidic (pH 3.3) and neutral (pH 7) media, observing higher amounts of volatile compounds under acidic conditions, as well as the presence of eugenol, probably in glycosidically bound form. For *T. bicolor*, esters such as ethyl acetate and ethyl benzoate were also identified, along with linalool [94]. This species is characterized by a fresh, fruity aroma due to lower esters, a floral note attributed to linalool and ethyl benzoate, and a fatty note associated with fatty acids.

It is important to note that the perception of aroma is the result of complex interactions among multiple compounds; therefore, establishing a relationship with a single volatile compound often does not represent the actual sensory profile.

### 3.4. Mucilage Applications

In recent years, there has been an increasing awareness of the environmental impact of reinserting agri-food byproducts into the industry. The multiple applicability of this species has been demonstrated, and several authors have catalogued *Theobroma* spp. as a promising fruit for commercialization within the biodiversity of the Amazon region [99]. The conventional cocoa processing techniques entail a fermentation process that is intrinsic to the natural characteristics of the mucilage that envelops the cocoa bean. This process is advantageous in enhancing the sensory attributes of the final product. However, research has indicated that partial pulping of cocoa beans is advisable, as it does not hinder the fermentation process and it promotes the quality of the beans. Consequently, a substantial portion of the mucilage can be recovered and reintegrated into the value chain for various applications.

Table 6 showed the production of foods derived from *T. cacao*, *T. grandiflorum* and *T. bicolor* mucilage.

A significant challenge in the commercialization of *T. cacao* mucilage is its limited shelf life, attributable to its high moisture content and fermentable sugars, which render it a substrate of considerable biotechnological interest [107]. Research conducted [55] evaluated the preservation of mucilage through the application of pasteurization and UHT treatments. It was observed that the latter exhibited a higher incidence of nonenzymatic browning reactions, which were attributed to the elevated temperatures (135 °C) employed. Nevertheless, the UHT treatment demonstrated greater effectiveness in reducing the total mold and yeast counts (<100 CFU/g), an effect that, when considered in conjunction with the low pH of the mucilage, could be considered a limiting factor for microbial proliferation. Meanwhile, Ref. [108] appraised the impact of single and double pasteurization on total polyphenol content, antioxidant capacity, and storage stability. The study concluded that double pasteurization facilitated a reduced loss of active compounds. In addition, it was reported that storage temperatures of 4 °C and 25 °C were suitable for preserving the quality of the mucilage. On the other hand, Ref. [109] evaluated untreated mucilage syrups formulated with palm sugar, cane sugar, and refined sugar. Despite being stored at a temperature of 5 °C, these products exhibited a limited shelf life of only 5 days, indicating the necessity of thermal processing. In conclusion, the significance of implementing suitable thermal processes to guarantee microbiological stability and prolong the shelf life of mucilage derived products is emphasized by these findings.

Furthermore, *T. cacao* mucilage has been extensively explored as a raw material for the production of fermented beverages, including craft beer [58], kombucha [35], cocoa wine [102], non-alcoholic beverages [104] and musts [100]. However, the efficacy of these applications require strict control of ethanol production, whose yield is primarily dependent on the initial sugar content such as sucrose, glucose, and fructose, as well as on the growth rate and metabolic activity of the microorganisms involved in fermentation. Beyond its incorporation into fermented products, mucilage has been used in the formulation of jellies, ice creams, and nectars [103], where it performs both sensory and techno-functional roles by providing sweetness, aroma, and structural stability due to its pectin content. Furthermore, it has been documented that these matrices possess the capacity to retain substantial levels of flavonoids, vitamin C, and antioxidant activity, thereby augmenting their nutritional and functional value [53]. In addition, mucilage has been evaluated for its potential as a substrate in bioethanol production [101] and cellulose synthesis [57].

Regarding *T. grandiflorum*, applications have been reported mainly toward the development of biomaterials, particularly the formation of biofilms combined with pectin and chitosan. These biofilms exhibited satisfactory mechanical properties, such as adequate tensile strength and low permeability, partially attributed to the plasticizing effect of the sugars present, highlighting their potential application in food packaging systems [105]. Likewise, drying and microencapsulation processes using maltodextrin and inulin have been explored to obtain mucilage powders rich in ascorbic acid and phenolic compounds, with a low degree of agglomeration [106]. Additional applications include its use in the formulation of prebiotic and probiotic yogurts, in which improvements in texture and rheological properties have been observed in yogurts produced from goat milk [99]. Moreover, *T. grandiflorum* mucilage has been evaluated as a substrate for the cultivation of *Lactobacillus rhamnosus*, whose fermentation leads to lactic acid production, a compound that has demonstrated beneficial effects against endotoxemia [61] as well as in ice cream formulations.

By contrast, reports on applications of *T. bicolor* mucilage remain limited; however, its use in jelly formulations through the addition of stabilizing agents has been documented, suggesting an incipient technological potential that requires further research and development for industrial valorization [32].

## 4. Technological and Industrial Challenges

*Theobroma* spp. represent a significant economic value in the global market, with *T. cacao* being the most relevant species compared to *T. grandiflorum* and *T. bicolor*, as its dry beans are primarily used in the chocolate industry. Worldwide, cocoa production exceeded 5 million tons in 2020/2021, and its market is projected to reach USD 67 billion by 2032 [110]. The production of dry beans generates agro-industrial residues such as husk, placenta, and mucilage, which together account for approximately 85% of discarded material, with mucilage representing 3–5%. However, the growing interest in the valorization of cocoa agro-industrial by-products has positioned mucilage as a low-cost ingredient with high technological potential for the food industry, as this by-product degrades rapidly during the processing of fresh beans and is generally discarded in agricultural fields, representing an opportunity for the development of new commercially valuable products and an alternative source of income for farmers. In *T. grandiflorum* and *T. bicolor*, mucilage may have a higher economic value than the beans, although data on its market remain limited due to its recent valorization.

While the European Food Safety Authority (EFSA) has classified it as a traditional ingredient suitable for human consumption [66], its development on an industrial scale remains limited by various technological and operational challenges. In particular, in the case of *T. cacao*, deficiencies have been reported in agricultural and manufacturing practices during the separation of the beans from the placenta, a process usually carried out in the field to facilitate transport to fermentation plants. These conditions lead to microbiological contamination of the mucilage. Furthermore, this initial handling triggers the activation of peroxidase enzymes, which compromises its stability and promotes uncontrolled biochemical reactions during fermentation, exacerbated by its high water and fermentable sugar content. In contrast, in *T. grandiflorum* and *T. bicolor*, the fruits are usually harvested and transported to the processing plant.

The greatest challenge in the industrialization of mucilage arises after its separation from the bean. This requires rapid conditioning without compromising its chemical, functional, and sensory characteristics to mitigate the risk of microbiological contamination and sensory degradation. These techniques have not yet been developed or designed on an industrial scale, as the reviewed literature has not identified the use of medium- or large-scale equipment, primarily for *T. bicolor*. However, *T. cacao* and *T. grandiflorum* have shown greater exploration due to their high economic value. The latter, in particular, benefits from the use of mucilage as a raw material, which is processed using small scale pulping machines and stored at low temperatures, reducing contamination and improving preservation. Consequently, the implementation of effective preservation strategies is essential. Therefore, specific research is required to develop and optimize scalable solvent extraction, stabilization and preservation processes (freezing, spray drying and freeze-drying) suitable for industrial and commercial applications, facilitating market access and supporting economic development and GDP growth in *Theobroma* producing regions.

The lack of technical criteria for the processing of value-added products constitutes a critical barrier to industrial scaling and the harmonization of regulatory frameworks intended to guarantee product safety, quality, and consistency.

## 5. Conclusions and Future Prospects

Theobroma spp. is native to Amazonian territories. The species *T. cacao*, *T. grandiflorum* and *T. bicolor* have demonstrated the most significant development since pre-Columbian times. The cacao bean *(T. cacao)* is an essential ingredient in the chocolate industry. However, recent research has explored the potential for innovating chocolate formulations by partially substituting *T. grandiflorum* and *T. bicolor* beans, which are collectively referred to as ‘Blend’. The growing development of the chocolate industry has led to the need to revalue its byproducts, generating renewed interest in *Theobroma* mucilage. In the case of *T. grandiflorum* and *T. bicolor*, the mucilage has traditionally been used as the primary product, with the beans being considered as byproducts.

The mucilage of *Theobroma* spp. has attracted interest due to its classification as a promising ingredient based on its valuable contents and availability. The mucilage contains a high concentration of sugars (sucrose, glucose and fructose), pectin, dietary fiber (hemicellulose and cellulose), and micronutrients (N, Ca and K). Furthermore, the presence of volatile compounds has been identified as a contributing factor to the floral aromas exhibited. From a functional perspective, the presence of mucilage is indicative of the high content of methylxanthines, polyphenols and organic acids (mainly citric and malic acid), which collectively confer a high antioxidant capacity.

The valorization of mucilage as a byproduct of the agro-industrial sector represents a strategic alternative for enhancing the economic and environmental sustainability of the *Theobroma* spp. production chain. This approach promotes the incorporation of mucilage as a functional ingredient, natural additive, substrate, and nutraceutical. However, agro-industrial waste intended for food production is subject to stringent regulatory requirements regarding environmental protection, safety, and quality. In view of the above, extracting mucilage in the harvest field carries a high risk of microbiological contamination and accelerated fermentation processes. Therefore, a comprehensive approach is required that combines technological innovation with compliance with international regulatory frameworks and specific regulations in each country.

The growing consumer preference for healthy diets has increased the demand for biologically active enriched foods. However, it has been proven that the application of uncontrolled heat treatments and prolonged exposure to the environment can have a significant impact on the functional properties and volatile composition of *Theobroma spp.* mucilage. In the current context, future prospects focus on the use of emerging technologies, such as freeze-drying, spray drying, UHT pasteurization and the incorporation of high hydrostatic pressures (HPP), with the aim of preserving functional compounds, minimizing bacterial load and efficiently conserving the sensory properties of mucilage. Due to its high sugar content, mucilage can be used as a fermentation substrate in the production of beer, kombucha, wine and spirits. At the same time, its structural properties derived from the presence of pectin make it an ideal stabilizer for the production of nectars, ice creams and jams. It is also important to highlight the biotechnological applications of the product in cellulose biosynthesis and biofilm production.

In this regard, the future prospects for the food industry focus on the development of new innovative products that preserve these compounds, ensuring not only their safety as well as the technological viability, process scalability, and compliance with legislation and directives on new foods derived from *Theobroma* spp. mucilage.

## Figures and Tables

**Table 1:** Morphological characteristics of the tree and fruit of Theobroma spp.

Characteristic	*T. cacao*	*T. grandiflorum*	*T. bicolor*
Production	n.d.	n.d.	Average of 40 pods per plant.
Pod morphology	Varies between elliptical, oblong, oblate, and orbicular shapes.	Oval to elongated berry.	Oblong or ovoid–lipsoid to subglobose fruit.
Peel	Hard and rough peel composed of epicarp, mesocarp, and endocarp.	Outer shell covered with a brown powdery layer.	Woody and rough shell.
Colour	According to variety and pollination: yellow, purple (wine red), and orange.	Brown.	Yellow to yellowish-brown.
Thickness, cm	n.d.	1–3	1.2
Length, cm	10–35	12–25	10–35
Diameter, cm	n.d.	10–12	9–15
Weight, kg	0.2–1	1–2	0.5–3
Groove	Prominent grooves (5–7 ridges)	No present	Prominently veined (4–6 ridges)
Beans	Ellipsoid to ovoid shape, 2–3 cm. A pod contains 30–45 beans.	Contains 20–50 beans surrounded by mucilage.	Arranged in 5 rows, surrounded by mucilage (1.6–3 cm by 1.4–2.5 cm).
Mucilage	White mucilaginous pulp with a sweet-sour taste.	White or creamy-yellow colour with a bittersweet taste.	Whitish to yellowish colour, strong odour and bittersweet flavour.
Ref.	[16,21,22]	[23,24]	[25,26]

n.d.: not detected.

**Table 2:** Percentage of co-products from various species within the genus Theobroma.

Specie	Common Name	Pod Shell	Grain	Mucilage	Husk	Placenta	Ref.
		%	%	%	%	%	
*T. Cacao*	Cocoa	68–79	14–20	3.5–5.30	1.4–2.2	0.75–3.2	[12]
*T. grandiflorum*	Cupuaçu	42–46	17–20	35–37	n.d.	n.d.	[28]
*T. bicolor*	Macambo	62.5	13.7	23.8	n.d.	n.d.	[29]

n.d.: not detected.

**Table 3:** Proximal composition, physical–chemical properties, sugars, structural polysaccharides and micronutrients of *Theobroma spp*. mucilage.

Specie	Proximal Composition (g/100 g) FW						Physical and Chemical Properties			Sugars (mg/g) DW				Structural Polysaccharides (%)			Micronutrients (mg/100 g)			Ref.
	Water	Ash	Lipids	Protein	Fibre	Carbohydrate	°Bx	pH	TEA	RS	Suc	Glu	Fruc	Pect	Hem	Cel	N	Ca	K	
*T. cacao*	85–94	0.4–0.9	0.8–1.5	0.3–0.6	0.47	15–18	14–17	3–4	18	172	35	69	72	0.5–1.2	0.2–3	0.7–0.9	52	6	27	[8,27,55,56,57,58,59]
*T. grandiflorum*	82–85	0.8–3.3	0.4–2.3	0.8–2.5	n.d.	9–11	12	3	60	89	108	24	36	n.d.	n.d.	n.d.	n.d.	6	34	[8,10,60,61]
*T. bicolor*	85–92	0.7–1.6	0.3	1.3–4.9	0.8–1.4	5–7	12	5–7	8	120	77	26	18	n.d.	n.d.	n.d.	n.d.	n.d.	n.d.	[8,62,63]

Bx: Brix, TEA: titratable acidity CA equivalent (mg/g), RS: reducing sugar, Suc: sucrose, Glu: glucose, Fruc: fructose, Pect: pectin, Hem: hemilose, Cel: cellulose, n.d.: not detected.

**Table 4:** Sample preparation and analysis parameters for the quantification of bioactive compounds in *Theobroma* spp. mucilage.

Specie	Sample	Sample Conditioning	Analysis	Method	Value	Ref.
*T. cacao*	Freeze-dried	pulp: juice: lyophilized ratio (1:0.87:0.16)	Total phenols	Folin–Ciocalteu, Absorbance (λ= 760 nm)	340 µg TAE/g	[8]
	Freeze-dried	Soxhlet (50 °C, 70% EtOH), ratio 1:20	Total phenols	Folin–Ciocalteu Absorbance (λ= 700 nm)	50 mg GAE/100 g	[75]
	Leached	Fermentation: National–Trinitario hybrid*: 4 days CCN-51: 6 days	Total phenols	Folin Ciocalteu Absorbance (λ= 760 nm)	National–Trinitario hybrid*:105 CCN-51: 72 Expressed in mg GAE/100 mL	[12]
	Leached	Fermentation: National–Trinitario hybrid*: 4 days CCN-51: 6 days	Total flavonoids	Extraction MetOH, reaction NaNO_3_, AlCl_3_, NaOH Absorbance (λ= 490 nm)	National-Trinitario hybrid*:37 CCN-51: 5 Expressed in mg CE/100 mL	[12]
	Leached	Fermentation: National–Trinitario hybrid*: 4 days CCN-51: 6 days	Content of flavan-3-ols	HPLC DAD (λ= 280 nm), Mobile phase: Phase A: CH_3_CN/H_2_O/HCOOH (99/0.8/0.2), isocratic mode. Phase B: CH_3_CN, gradient mode (5% to 100% in 67 min).	National–Trinitario hybrid*: Procyanidin B1: 2.6 Procyanidin B2: 3.5 Procyanidin C1: 1.7 Epicatechin: 1.4 Catechin: 3.5 CCN-51 Procyanidin B1: 0.6 Procyanidin B2: 0.9 Procyanidin C1: 0.6 Epicatechin: 0.7 Catechin: 0.3 Expressed in mg/100 mL	[12]
	Leachate	Fermentation: National–Trinitario hybrid*: 4 days CCN-51: 6 days	Methylxanthines	HPLC DAD (λ= 273 nm) Mobile phase: MetOH: water (25%)	National–Trinitario hybrid* Caffeine: 0.9 Theobromine: 2.7 CCN-51 Caffeine: 0.1 Theobromine: 0.5 Expressed in g/100 mL	[12]
	Leachate	Fermentation: (a) National–Trinitario hybrid*: 4 days (b) CCN-51: 6 days	Antioxidant capacity	ABTS/FRAP/ORAC	ABTS: (a) 8.5, (b) 4.6 FRAP: (a) 7.9, (b) 3.4 ORAC: (a) 1.3, (b) 1.3 Expressed in μM TE/mL	[12]
	Fermented	Time fermentation: 24, 48 and 72 h	Antimicrobial capacity	-	*Lactic acid strain* *Salmonella* sp.: CCN51-5 (58%) and CCN51-4 (54%) *Escherichia coli*.: CCN-5 (59%) y MM1-2 (57%)	[76]
	Extract	Mucilage purification with 95° ethanol	Antibacterial activity	Distribution on paper disc	Extraction yield: 0.4% Concentration of 4000 µg/mL No zone inhibition for: *Staphylococcus aureus* *Escherichia coli* *Pseudomonas aeruginosa*	[77]
*T. grandiflorum*	Freeze-dried	Pulp: juice: freeze-dried ratio (1 g:0.87 mL:165 mg)	Total phenols	Folin–Ciocalteu, Absorbance (λ= 760 nm)	226 µg TAE/g	[8]
	Fresh	Extraction: (a) EtOH (80%) (b) MetOH (70%) (c) Acetone (70%)	Total phenols	Folin-Ciocalteu	(a) 52 (b) 47 (c) 66 Expressed in mg GAE/100 g	[78]
	Fresh	Extraction: (a) EtOH (80%) (b) MetOH (70%) (c) Acetone (70%)	Flavonoids	AlCl_3_ method Na_2_CO_3_ (5%), AlCl_3_ (10%) NaOH and H_2_O	(a) 4.1 (b) 0.6 (c) 3.1 Expressed in mg QE/100 g	[78]
	Fresh	Extract: MetOH: H_2_O (50:50), pH 2	Total phenols	Folin–Ciocalteu Absorbance (λ= 725 nm)	40 mg GAE/100 g	[79]
	Extract	Extraction MetOH: H_2_O (7:3)	Total phenols	Folin–Ciocalteu Absorbance (λ= 760 nm)	154-171 mg GAE/100 g	[50]
	Extract	Extraction MetOH: H_2_O (7:3)	Total flavonoids	AlCl_3_ method Na_2_CO_3_ (5%), AlCl_3_ (10%) NaOH and H_2_O	142- 147 mg C/100 g	[50]
	Fresh	100 mg sample in 1 mL MetOH: H_2_O: HCOOH	Content of flavan-3-ols	HPLC-UV (λ= 280 nm) Mobile phase: (A) H_2_O/HCOOH (99:1) (B) CH_3_CN Standard: catechin and quercetin	catechin: 11–19 quercetin: 5–7 Expressed in mg/100 g	[50]
	Extract	Extraction MetOH: H_2_O (7:3)	Antioxidant capacity	ABTS/DPPH/ORAC	ABTS:91–97 DPPH: 4 ORAC: 50–68 Expressed in μM TE/g	[50]
*T. bicolor*	Pasteurised	Pasteurised at 90 °C, frozen at −4 °C	Total phenols	Folin–Ciocalteu	162 mg GAE/100 g	[26]
	Freeze-dried	Extract: 20 mg sample with 500 µL of 80% MetOH with 0.1% HCl	Total phenols	HPLC DAD.UV-VIS (λ= 273 nm) Mobile phase: MetOH: water (25%)	245 mg GAE/100 g	[54]
	Freeze-dried	Extract: 20 mg sample with 400 µL MetOH	Antioxidant capacity	DPPH/ABTS	DPPH: 2 ABTS: 3 Expressed in mM TE/100 g	[54]
	Pasteurised	Pasteurised at 90 °C, frozen at −4 °C	Antioxidant capacity	DPPH Absorbance (λ= 550 nm)	2 mg TE/100 g	[26]

λ: wavelength, EtOH: ethanol, MetOH: methanol, Na_2_CO_3_: sodium carbonate, NaOH: sodium hydroxide, AlCl_3_: aluminium chloride, ABS: absorbance, H_2_O: distilled water, HCOOH: formic acid, CH_3_CN: acetonitrile, ABTS: 2,2′-azino-bis(3-ethylbenzothiazoline-6-sulfonic acid), DPPH: 2,2-diphenyl-1-picrylhydrazyl, FRAP: Ferric Reducing Antioxidant Power, ORAC: Oxygen Radical Absorbance Capacity, HPLC-DAD: High-Resolution Liquid Chromatography with Diode Array Detector, TAE: tannic acid equivalents, GAE: gallic acid equivalents, QE: quercetin equivalents, TE: trolox equivalents.

**Table 5:** Volatile compounds present in *Theobroma spp.* mucilage.

Specie	Matrix	Type of GC	Extraction or Concentration	Odorant Compound	Concentration/FD/RI	Odorant Quality	Ref.
*T. cacao*	Fresh	GC-MS	Headspace solid-SPME	2-Pentanal Linalool 2-Pentanone 2-methyl-3 Buten-2-ol	15–41% 2–36% 5–26% 2–15%	almonds, malt flowery fruity herbal	[22]
	Fresh	GC-O GC-MS/O SBSE-GC-MS/O HS-SPME GC-O/MS	SAFE SBSE Headspace solid-SPME	Trans-4,5-epoxy-(E)-decenal 2- and 3-methylbutanoic acid 3-(methylthio)propanal 4-vinyl-2-methoxyphenol Linalool	512–1024 128–1024 64–512 128–512 32–512	metallic rancid, cheesy cooked potato-like smoky, clove-like flowery	[31]
	Fresh/Pasteurised/UHT	GC-O GC-MS/O SBSE-GC-MS/O HS-SPME GC-O/MS	SAFE SBSE Headspace solid-SPME	2,3-pentanedione 3-(methylthio)propanal 1-pentanol Linalool (E, E)-2,4-nonadienal (E, E)-2,4-decadienal 5-methylguaiacol Trans-4,5-epoxy-(E)-2-decenal 2,5-dimethylphenol	64–256 64–256 <1–769 128–512 32–≥1024 512–≥1024 64–256 64–512 32–512	butter-like cooked potato-like pungent flowery deep fried, fatty deep fried, fatty smoky, vanilla-like metallic smoked ham-like, medical	[55]
	Fresh	GC-O	SAFE distillation	2-Phenylethanol trans-4,5-epoxy-(E)-2-decena γ-Nonalactone Linalool (E, E)-2,4-decadienal (E,E)-2,4-nonadienal 2- and 3-methylbutanoic acid 3-(methylthio)propanal	2–32 32–128 1–16 1–8 4–32 8–16 2–32 2–32	flowery, sweet metallic coconut-like citrus-like, floral tallowy, fatty fatty, green rancid cooked potato	[91]
*T. grandiflorum*	Fresh	GC-FID GC-MS	Continuous liquid–liquid extraction	Ehyl hexanoate Linalool Ethyl 3-hydroxyhexanoat α-Terpineol Ethyl butanoate Butyl acetate	359–1253 104–986 520 89–440 381–389 7–440 Expressed in μg/kg	n.d.	[19].
	Frozen	GC-FID GC-MS	Volatile compounds from the headspace	Ethyl butanoate Ethyl hexanoate Hexadecanoic acid	42.2 21.19 12.5 Expressed in % area	n.d.	[92]
	Fresh	HS-SPME-GC-MS	headspace vial-SPME	linalool α-terpineol *trans*-4-methoxythujane 2-methylbutyl butanoate 3-methylbut-2-enoic acid 2-methylpentyl ester 2-methylpropyl hexanoate	25.35 28.75 25.44 23.43 - - 27.44 Expressed in retention time	n.d	[93]
*T. bicolor*	Fresh	GC-FID	liquid–liquid extraction headspace vial-SPME	Ethyl acetate Linalool Ethyl benzoate	3.23 2.14 1.24 Expressed in mg/kg	n.d.	[94]

GC-MS: gas chromatography–mass spectrometry, Gas Chromatography–Olfactometry, SPME: solid-phase microextraction, SBSE: Stir Bar Sorptive Extraction, SAFE: Solvent-Assisted Aroma Evaporation, DCM: dichloromethane, EI: electron impact, FID: flame ionization detector, OPD: odor detection port, n.d: not detected.

**Table 6:** Applications of *Theobroma* spp. mucilage.

Species	Type of Application	Parameter Studied	Selected Conditions	Ref.
*T. cacao*	Concentrated pulp	Pasteurised (80 °C for 30 s) UHT (135 °C for 30 s). Cooled to 0 °C.	Colour: L* value decreases a_w_: 0.98 POD: absent Total yeast and mould count: <100 cfu/g.	[55]
	Substrate for Green Tea Kombucha	Type: national cocoa and CCN-51. Concentration sugar: 40, 60, 80 and 100 g/L	National cocoa and sugar (40 g/L) Sensory analysis: acceptable taste, texture and sweetness	[35]
	Application as an adjunct in beers	Concentration: 10, 30 and 49%.	30% mucilage Ethanol production: S-23 yeast (0.5 gL/h) and S-04 yeast (0.6 gL/h). 22 °C: 60 h: 75%	[58]
	Fermentation substrate by *Saccharomyces cerevisiae*	Residual must: 19 °Brix, pH 5, K2S2O5 (200 mg/L). Yeast strain: *S. cerevisiae AWRI726*: activation in YEPD medium for 24 h at 28 °C. Fermentation: 20 °C for 240 h.	144 h: ethanol 14% *v*/*v*, biomass 6 g/L Maximum yield: 24 h Ethanol yield: 0.4 g/g Volumetric productivity: 0.2 g/L*h Cell growth rate: 0.3.	[100]
	Bioethanol	Activation: 1.5 g of yeast with 50 mL of mucilage (70 °C for 15 min. Fermentation: 3–7 days.	pH: 3–4 Reducing sugar: 7.8–8.3% Ethanol: 5% (classified as low)	[101]
	Cellulose biosynthesis	Culture medium: xylinus, sterilized cacao mucilage exudate, supplemented (5 g/L peptone, 5 g/L yeast extract and 2.7 g/L sodium citrate), diluted/supplemented (1:2 exudate: water, 5 g/L peptone, 5 g/L yeast extract and 2.7 g/L sodium citrate)	Diluted/supplemented (1:2 exudate: water) + N, cellulose biosynthesis yield: 0.6 to 13.2 g/L. Biosynthesis rate: 0.04 g/L*h. Production scale from 30 mL to 15 L.	[57]
	Fruit wine	*S. cerevisiae* (*CA116*, *CA1162* and *CA1183*). Glucose (10.0 g/L); KH_2_PO_4_ (4.5 g/L); (NH_4_)2SO_4_ (3.0 g/L); yeast extract (1.0 g/L); MgSO_4_Æ7H_2_O 0.25 g L)1 CaCl_2_ (0.25 g/L) pH 5.0 Incubation Temperature: 18, 22 and 25 °C	CA1183. Ethanol: 12% (*v*/*v*). Ethanol yield: 46% (18 °C, 22 °C, 25 °C) Glycerol: 9 g/L (18 °C, 22 °C, 25 °C)	[102]
	Mucilage jelly	National and CCN-51 cocoa mucilage. Sugar concentration at 35.40 and 45%. pH: 0.5%	CCN-51 (40% sugar + 0.5% pectin) °Brix: 65, pH 3.4, Acidity 1%, Moisture 37%, Protein 0.6%	[103]
	Non-alcoholic beverage	Pasteurized pulp inoculated with *Laetiporus persicinus* Dilution: 185 mL H_2_O: 20 g cocoa mucilage Fermentation: 150 rpm at 24 °C Fermentation harvest: 12, 24, 48, 60 and 72 °C	Fermentation time: 48 h Beverage aroma: (R)-linalool, 5-butyl-2(5H)-furanone, (E)-nerolidol and 2-phenylethanol	[104]
*T. grandiflorum*	Edible films	Molding method Non-nanostructured films (50% water, 47% *T. grandiflorum* pulp and 2.3% pectin) and nanostructured films (50% chitosan, 47% *T. grandiflorum* pulp and 2.3% pectin).	**Non-nanostructured films:** Tensile strength (2 and 3% pectin): 15 mPa and 23 mPa. Permeability: 2.5 g mm/kPa h m^2^ **Nanostructured films:** Tensile strength (2 and 3% pectin) 25 mPa and 30 mPa Permeability: 1.9 g mm/kPa h m^2^	[105]
	Spray-dried	Dilution ratio: pulp/water (1:1.25) Wall material: maltodextrin (8.2 g-42.8 g/100 g) Inulin (17.2–50.8%). Drying parameters: (air temperature at 118 and 198 °C, flow rate of 7.5 mL/min and suction rate at 100%)	Optimal parameters: °T. inlet (185 °C), inulin (44%), maltodextrin (35%) Process yield (53%) Ascorbic acid encapsulation efficiency: 96% Phenol encapsulation efficiency: 62%.	[106]
	Added to goat’s milk yoghurt	Treatment: natural yoghurt, probiotic yoghurt (*L. acidophilus LA-5*), prebiotic yoghurt (inulin), symbiotic yoghurt, yoghurt with pulp, probiotics with pulp. Fermentation: 43 ± 2. pH: 4.5.	Storage 28 days: Probiotic count: ≥7 log CFU/g LA-5 Colour: ≤ (L* 90, a* 2 and b* 5) L* increase, a* increase, b* decrease. Apparent viscosity: yoghurt with pulp, probiotics with pulp behavior (450–500 mPa*s), viscosity reduces from day 0 to 28. Texture: Firmness (≥18 g), consistency (≥98 g/s) and cohesion (≥−32 g)	[99]
	Fermented substrate *Lacticaseibacillus rhamnosus*	Concentration: 120 mg/mL pH: 6 Pre-inoculum of *L. rhamno-sus ATCC 9595* (37 °C, 120 RPM and 24 h) Bacterial suspension: OD at 600 nm of 1. Inoculation: 1 mL inoculated into the juice (120 RPM, 48 h) DCCR optimization: Inoculum density (0.77–2.33 DO 600 nm) Pulp concentration: 135.25–324.75 mg/mL)	Lactic acid: 1.3–5.7 g/L. Growth: 6–9 CFU/mL pH: 3.7–4.9 Growth/pH: 1.4–2.5 Growth/[La]: 1.5–11.3 Substances differentially present with fermentation by *L. rhamnosus*: theobromine, vanillic acid glycoside, epicatechin.	[61]
*T. bicolor*	Nectar	Concentration 25, 50, 75 and 100%. With CMC and without CMC.	Viscosity (70–815), turbidity (54–165), °Brix (13–18), pH (3.5–4.3) and titratable acidity (0.4–0.7) Colour: L* 38, a* −8 and b* −10. 25% with CMC.	[32]

## Data Availability

No new data were created or analyzed in this study. Data sharing is not applicable to this article.
